# Muscle oxygenation levels in smokers and non-smokers during exercise: insights from a university-based study

**DOI:** 10.1007/s00421-025-05829-8

**Published:** 2025-06-14

**Authors:** Buğra Kerget, Büşranur Taşkin, Alperen Aksakal, Hatice Beyza Özkan, Elif Yilmazel Uçar

**Affiliations:** 1 Department of Pulmonary Diseases, Ataturk University School of Medicine, 25240 Yakutiye, Erzurum, Turkey; 2 Ataturk University School of Medicine, 25240 Yakutiye, Erzurum, Turkey; 3 Chest Disease Department, Yakutiye Medical Research Center, 25240 Yakutiye, Erzurum, Turkey

**Keywords:** Near-infrared spectroscopy, Smoking, 6MWT, Vastus lateralis, Exhaled CO

## Abstract

**Background:**

Today, as the age of smoking is decreasing, awareness of the harms of tobacco is still not at a sufficient level among young people. In our study, we aimed to compare the muscle oxygenation levels between students who smoke and those who do not smoke after the training on the harms of tobacco products.

**Methods:**

A total of 40 smokers and 30 non-smokers, aged 23 and studying at our university, participated between November 2024 and December 2024. Vastus lateralis muscle oxygenation was measured during the six-minute walking test (6MWT).

**Results:**

In smokers, exhaled CO, pulse at the beginning and end of 6MWT, Borg dyspnea, and fatigue scores were significantly higher than in non-smokers (*p* < 0.001). Maximum and minimum SmO_2_ levels measured at the beginning and end of 6MWT were lower in smokers (*p* < 0.001), while changes in SmO_2_ were greater in smokers (*p* < 0.001). Smoking level negatively correlated with maximum and minimum SmO_2_ and positively with ΔSmO_2_ (*R* = − 0.82, *p* < 0.001; *R* = 0.83, *p* < 0.001; *R* = 0.79, *p* < 0.001). Similarly, exhaled CO negatively correlated with maximum and minimum SmO_2_ and positively with ΔSmO_2_ (*R* = − 0.83, *p* < 0.001; *R* = 0.84, *p* < 0.001; *R* = 0.78, *p* < 0.001).

**Conclusion:**

Smoking causes a decrease in muscle oxygenation depending on the amount smoked. We believe that this cumulative burden may be a precursor to comorbidities that develop in later ages.

## Introduction

Smoking plays a vital role in the development of cardiovascular and pulmonary diseases associated with exercise intolerance. In addition to these diseases, smoking also plays a crucial role in the development of exercise intolerance (Morse et al. 2008). While comorbidities that develop due to smoking can show their effects more concretely at later ages, young individuals, unfortunately, have insufficient awareness of this issue.

Carbon monoxide (CO) is one of the main components of cigarette smoke. CO can have 200–250 times the affinity for hemoglobin compared to oxygen. This leads to various mechanisms affecting oxidative energy production in muscle tissue (Carrola et al. 2023); (a) the high-affinity binding of CO to the iron of the heme molecule leads to insufficient oxygen transport to the tissues, and (b) CO causes the oxygen dissociation curve to shift to the left. This situation leads to oxygen being able to leave the tissues at lower PaO_2_ pressure, and (c) CO binds to myoglobin in the muscle tissue, forming carboxy-myoglobin. This situation causes the inactivation of myoglobin, which is found as an oxygen reservoir in the muscle tissue; (d) CO interacts with cytochrome c oxidase, which is involved in energy production by oxidative means, and causes this molecule to be inactivated. These effects of CO in the body cause exercise intolerance in individuals and manageable muscle cramps (Decker et al. 2021, 2023).

Studies evaluating the effects of smoking on skeletal muscle have shown that fatigue resistance is lower in smokers than in non-smokers. In addition, the fatigue resistance in the muscles of COPD patients who quit smoking was equivalent to that of the control group, whose age and daily activities were similar. This shows that the effects of smoking on muscle fatigue are reversible (Bruce and Bruce 2006). In another study evaluating the effect of cigarette consumption on exercise intolerance, the increase in COHb levels caused exercise intolerance in the vastus lateralis muscle in the acute period. However, in the chronic period, the effect of CO on the mitochondrial system was more effective in causing exercise intolerance (Degens et al. 2024).

In our study, we aimed to compare the change in vastus lateralis muscle oxygenation during the 6-min walking test in young patients who are active smokers with those who have never smoked. We aimed to raise awareness about the harms of smoking in young people who have no idea about the effects on muscle oxygenation levels and whose smoking age is decreasing.

## Materials and methods

### Study design

Our study included 23-year-old active smokers who agreed to participate in our research and studied at Atatürk University between November 2024 and December 2024. The control group included students continuing their student life at our university and meeting our inclusion criteria—all patients who participated in the trial provided written informed consent. The study was developed and conducted in compliance with the ethical principles outlined in the Declaration of Helsinki, and the local ethics committee approved the study protocol.

### Study population

A 1-h lecture is given every month at our university to provide information about the harms of tobacco use. A total of 70 students, 40 of whom were active smokers and 30 of whom had never smoked, were included in our study after the lecture. The individuals included in our study were confirmed to have no obstructive pulmonary pathologies by performing pulmonary function tests (PFT) before the study. Individuals with active upper or lower respiratory tract infections, individuals with physical disabilities that prevented them from performing pulmonary function tests and six-minute walk tests (6MWT), patients who were followed up for acute coronary syndrome in the last month, those with obstructive pulmonary pathologies, and individuals who regularly perform sports activities that will increase lower extremity muscle strength and oxygenation were not included in our study.

#### PFT application

Age, height, and weight were measured before the respiratory function test. Before the test, care was taken not to smoke for 24 h, not to drink alcohol for 4 h, not to do heavy exercise for 30 min, not to wear clothing that restricts chest and abdominal movements, and not to eat a heavy meal for 2 h. BTPS correction was performed according to room air and barometric pressure. The technician explained the maneuver to the patient. The patient had three acceptable spirograms. Tests that met the respiratory function test reproducibility and acceptability criteria published by ATS/ERS in 2019 were included in the study (Graham et al. 2019). The lower limit of normal parameters determined for the healthy population was calculated and presented on the spirometry device by per this report's criteria. Spirometry was performed by the same technician using the Plusmed MIR SpiroLab III device.

#### Exhaled CO measurement and heaviness of smoking index (HSI)

Exhaled CO levels were measured using a Pico^™^ Smokerlyzer^®^ (Bedfont Scientific LTD, Kent, UK). The patients were instructed to inhale deeply, hold for 15 s, and slowly exhale until their lungs were completely empty. Exhaled CO was measured simultaneously in individuals who completed the PFT test. In the HSI score measurement, each question was scored between 0 and 3 points according to the time the students smoked their first cigarette after waking up (first 5 min: 3 points, 6–30 min: 2 points, 31–60 min: 1 point, after 60 min: 0 points) and the number of cigarettes smoked per day (less than 10: 0 points, 11–20: 1 point, 21–30: 2 points, more than 31: 3 points).

#### 6MWT and fingertip saturation measurement

Before the test, the patients rested for at least 15 min at the beginning of the 30-m track where the test would be performed, and their fingertip saturations were measured and recorded. The patients determined their speed and walked as fast as possible in a straight corridor for 6 min under the supervision of a physician. In any situation, such as excessive fatigue, shortness of breath, or palpitations during the test, the test was terminated without putting the patient at risk. At the end of the test, the patient was seated back in the chair, and the test values were recorded. Before and after the test, the patients' Borg dyspnea scores were calculated, with 0 being none at all and 10 being very severe (Enright 2003).

#### Application of near-infrared spectroscopy (MOXY^®^ monitor)

Near-infrared spectroscopy (NIRS) (MOXY, Minnesota, USA) measured muscle oxygenation. NIRS was placed on the right vastus lateralis region of the individuals included in the test. The device, which started recording at the beginning of the 6-min walking test, continued recording until the end. MOXY measures the ratio of oxyhemoglobin concentration to total hemoglobin concentration in the muscle in real-time and reports this as a percentage shown as muscle oxygen saturation or muscle oxygenation (SmO_2_). The collected data are stored as.csv files in the device's internal memory. All data were transferred to a Microsoft Excel file via Bluetooth connection with the device. Muscle oxygenation values obtained at the beginning and end of the test were included in the analysis.

### Statistical analysis

Analyses were performed using IBM SPSS Statistics version 27.0 software (IBM Corp, Armonk, NY). Pearson’s Chi-square test and Mann–Whitney *U* test were used for between-group comparisons of normally and non-normally distributed numerical data, respectively. An independent sample t test was used to compare parameter values of smoking and non-smoking groups. Relationships between two quantitative variables were examined using Pearson correlation analysis if normally distributed and Spearman correlation analysis if non-normally distributed. *p* values < 0.05 were considered statistically significant.

## Results

All patients included in our study were 23 years old. Twenty four of those in the smoker group and 18 in the non-smoker group were male. The percentage of males in both groups was observed to be 60. The students included in the study had no known chronic diseases.

Smoking, exhaled CO levels, heaviness of smoking index, pulmonary function test, Borg dyspnea, fatigue levels at the beginning and end of 6MWT, and muscle oxygenation levels of the students in the study are shown in Table 1. Accordingly, exhaled CO, pulse at the beginning and end of 6MWT, Borg dyspnea, and fatigue scores were statistically higher in smokers than in non-smokers (*p* < 0.001 for all). It was observed that maximum and minimum SmO_2_ levels measured at the beginning and end of 6MWT were lower in smokers than in non-smokers (*p* < 0.001 for all). SmO_2_ levels that changed at the beginning and end of 6MWT were higher in smokers than in non-smokers (*p* < 0.001).

**Table 1:** Comparison of values measured at the beginning and end of pulmonary function test, his, and 6MWT between smoker and non-smoker groups

	Smokers (*n* = 40) Mean ± SD	Non-smokers (*n* = 30) Mean ± SD	*p*
Cigarette (pack/year)	8.6 ± 5.1	–	N/A
Exhaled CO (PPM)	9.8 ± 3.7	2.6 ± 0.7	< 0.001
Pulse at start of 6MWT	108.4 ± 12.6	82.1 ± 7.2	< 0.001
Borg dyspnea score at start of 6MWT	0.8 ± 0.8	0.2 ± 0.1	< 0.001
Fatigue level at start of 6MWT	1.8 ± 1.3	0.4 ± 0.6	< 0.001
SO2 at start of 6MWT	96.1 ± 0.7	95.9 ± 0.7	0.21
Pulse at end of 6MWT	145.6 ± 14.3	105.3 ± 7.3	< 0.001
Borg dyspnea score at end of 6MWT	3.1 ± 1.9	0.5 ± 0.5	< 0.001
Fatigue level at end of 6MWT	4.2 ± 1.5	1.3 ± 0.6	< 0.001
SO2 at end of 6MWT	96 ± 1.1	95.6 ± 0.5	0.08
6MWT distance (meter)	624.2 ± 48.6	634.3 ± 47.4	0.44
Maximum SmO_2_	62.8 ± 9.9	80.5 ± 2.5	< 0.001
Minimum SmO_2_	46.4 ± 12.6	73.6 ± 3.3	< 0.001
ΔSmO_2_	16.7 ± 4.8	6.8 ± 2.1	< 0.001
HSI	3.7 ± 1.8	–	N/A
FVC (liter)	4.32 ± 0.78	4.29 ± 0.9	0.78
FVC (%)	98.1 ± 11.2	97.6 ± 12.5	0.75
FEV1 (liter)	3.8 ± 0.6	3.7 ± 0.7	0.82
FEV1 (%)	84.1 ± 5.4	83.9 ± 5.5	0.83
FEV1/FVC (%)	82.3 ± 3.3	81.7 ± 4.4	0.83

*CO* carbon monoxide, *6MWT* six-minute walking test, *HSI* heaviness of smoking index, *FVC* forced vital capacity, *FEV1* forced expiratory volume in 1 s

**Fig. 1:**
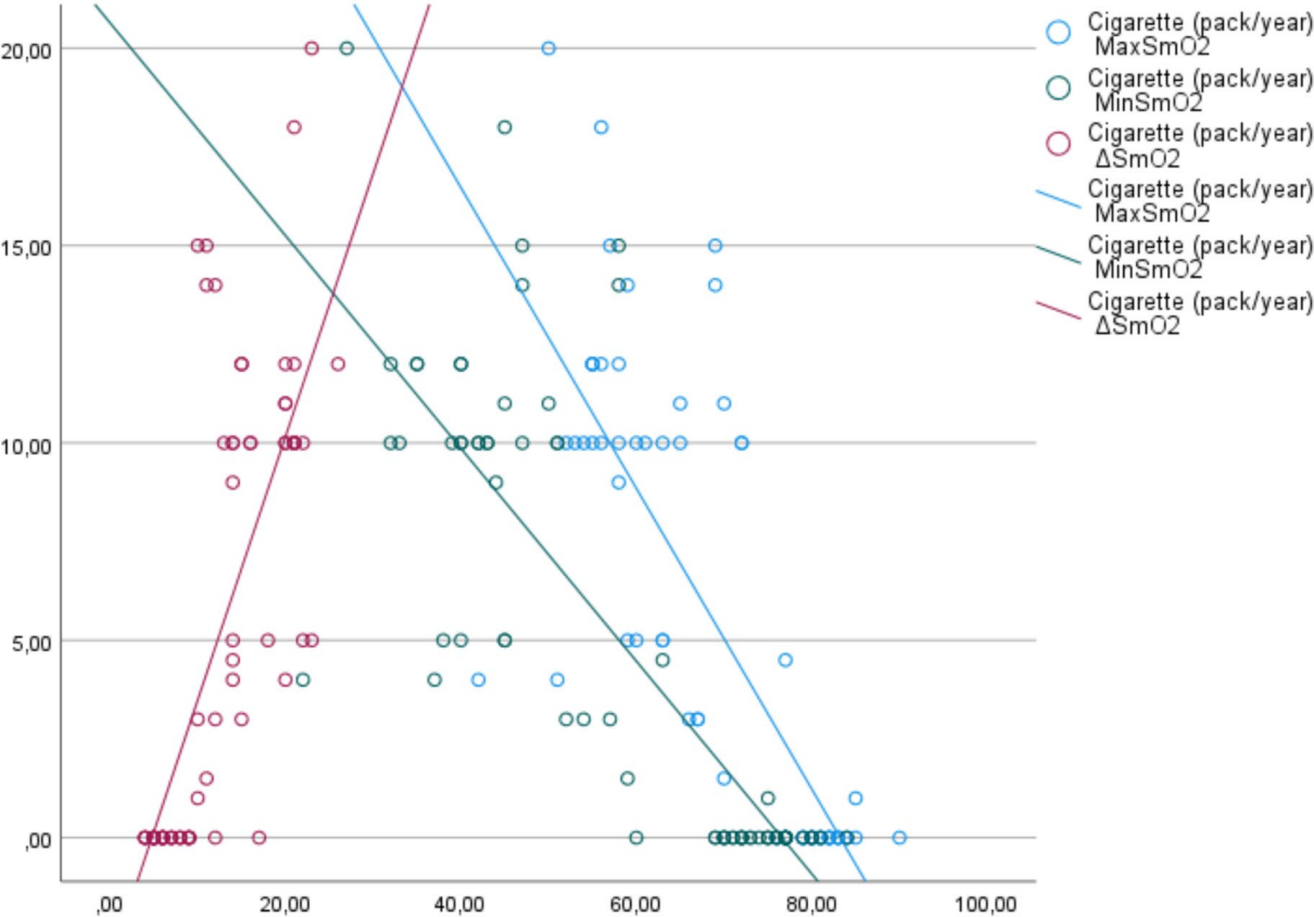
Correlation analysis of cigarette consumption and muscle oxygenation levels

**Fig. 2:**
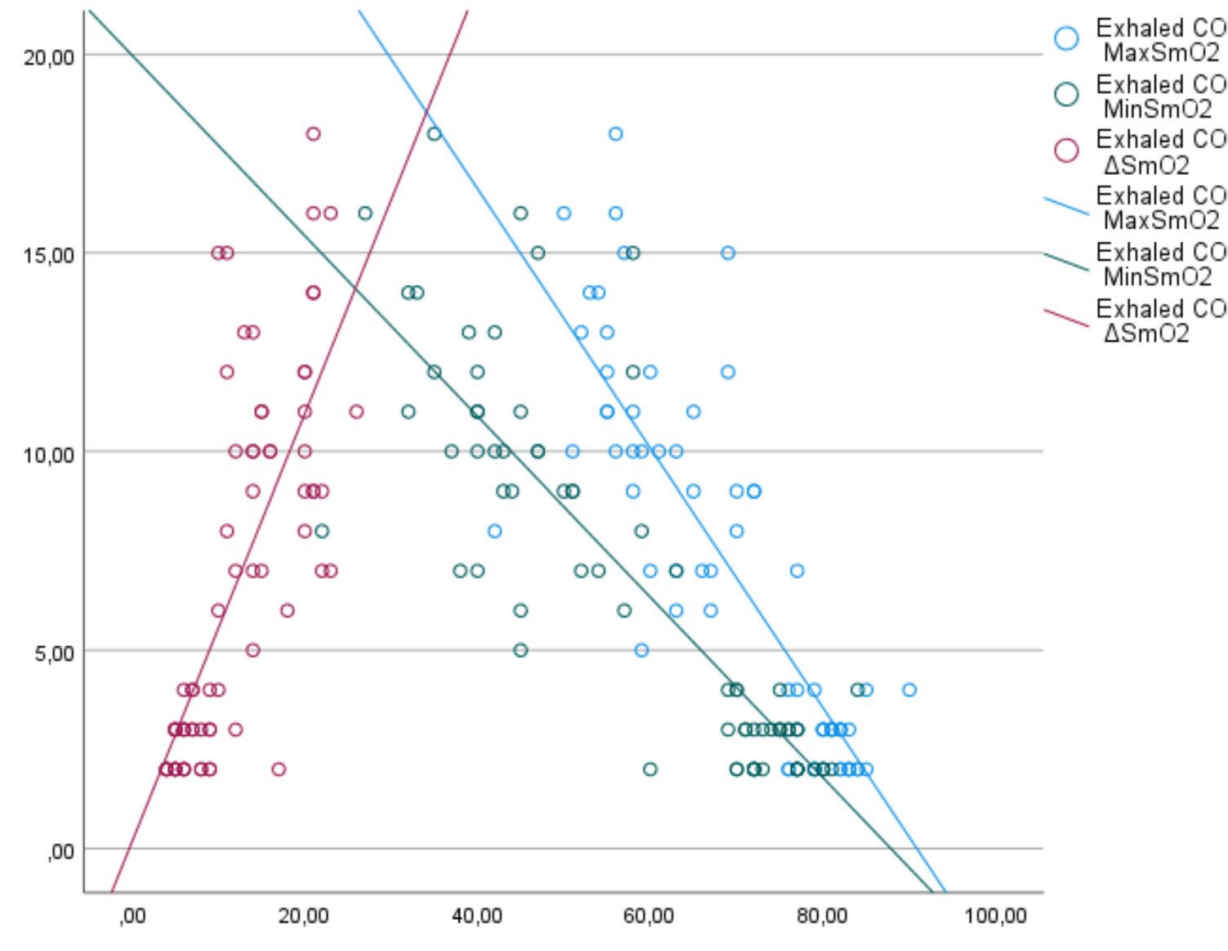
Correlation analysis of muscle oxygenation levels with exhaled CO

In the correlation analysis of the measured parameter values, a positive correlation was observed between smoking level and exhaled CO level and Borg dyspnea score at the beginning and end of 6MWT (*R* = 0.925, *p* =  < 0.001, *R* = 0.57, *p* =  < 0.001, *R* = 0.72, *p* =  < 0.001, respectively). Correlation analysis between smoking levels and maximum, minimum, and ΔSmO_2_ is shown in Fig. 1. Accordingly, a negative correlation was observed between smoking level and maximum and minimum SmO_2_, while a positive correlation was observed between ΔSmO_2_ (*R* = − 0.82, *p* =  < 0.001, *R* = 0.83, *p* =  < 0.001, *R* = 0.79, *p* =  < 0.001, respectively). Correlation analysis between exhaled CO and maximum, minimum, and ΔSmO_2_ is shown in Fig. 2. Accordingly, a negative correlation was observed between exhaled CO and maximum and minimum SmO_2_, and a positive correlation was observed between ΔSmO_2_ (*R* = − 0.83, *p* =  < 0.001, *R* = 0.84, *p* =  < 0.001, *R* = 0.78, *p* =  < 0.001, respectively). In addition, a negative correlation was observed between HSI level and maximum and minimum SmO_2_, and a positive correlation was observed between ΔSmO_2_ (*R* = − 0.91, *p* =  < 0.001, *R* = 0.9, *p* =  < 0.001, *R* = 0.79, *p* =  < 0.001, respectively).

At the end of the 6MWT, cramps and muscle pain in the gastrocnemius region were observed in 14 (35%) of the smoking students and 4 (13.3%) of the non-smoking students. In the statistical analysis, it was observed that cramps and muscle pain observed in the smoking students were statistically higher than in the non-smoking students (*p* =  < 0.001).

## Discussion

Our study observed that the vastus lateralis muscle oxygenation before and after the exercise test was lower in students who smoked compared to students who did not smoke. It was also observed that the decrease in muscle oxygenation with exercise was higher in students who smoked. The muscle oxygenation level was observed to be inversely correlated with the smoking level and exhaled CO level. It was also observed that muscle cramps and pain with exercise were higher in students who smoked compared to students who did not smoke.

Smoking is one of the most important causes of obstructive pulmonary pathologies, lung cancer, and recurrent lower respiratory tract infections. It is estimated that approximately 6 million people have died from diseases caused by smoking. While toxic gas inhalation due to smoking causes direct airway damage, the deterioration in tissue oxygenation and endothelial dysfunction indirectly affect organs outside the lungs (Sethi and Rochester 2000; Messner and Bernhard 2014; Golbidi et al. 2020).

A study conducted on middle-aged individuals without airflow limitation showed that individuals who smoked had lower exercise capacity compared to non-smokers (Mesquita et al. 2015). In addition, muscle tissue atrophy and increased glycolytic activity have been observed in smokers without any deterioration in lung function. It has been stated that this change in body composition may be the reason why anxiety and depression are more common in smokers (Pitsavos et al. 2005).

In our study, we ensured that the groups were homogeneous regarding age and gender to eliminate the effect of age and gender on muscle oxygenation. Although fingertip oxygen saturations were similar to those of non-smokers at the beginning and end of the exercise test in smokers, higher levels of Borg dyspnea, fatigue, and pulse were observed. However, smokers' muscle oxygenation levels were lower than those of non-smokers at the beginning and end of the test. This situation can be evaluated both due to the endothelial dysfunction that smoking causes directly in the tissues and the binding of CO to hemoglobin with a higher affinity than oxygen. The inverse correlation between smoking level and exhaled CO level and vastus lateralis muscle oxygenation can be considered as confirming this situation. Low muscle oxygenation is among the important causes of both fatigue and dyspnea in individuals (Kerget et al. 2024). Although similar pulmonary function test parameters were observed in students who smoked and those who did not, the fact that dyspnea and fatigue were more prevalent may be considered a result of impaired muscle oxygenation. The decrease in muscle oxygenation levels may have caused symptoms of muscle cramps and pain to occur in students who smoked at the end of the 6MWT. It was observed that the HSI level, which was made according to the number of cigarettes and the duration of smoking the first cigarette after waking up, correlated better with maximum and minimum muscle oxygenation. This situation can be evaluated as the shorter the duration of smoking the first cigarette after waking up, the higher the negative effect on muscle oxygenation level.

The vastus lateralis muscle was selected in our study, because it was most associated with exercise level and was easy to measure with the NIRS method. However, measuring muscle oxygenation levels simultaneously with several muscle groups during exercise may contribute to the confirmation of our study's results.

In conclusion, tobacco and tobacco products pose a significant threat to our youth today, where the age of starting to smoke has decreased. Although the incidence of smoking-related comorbidities is low in young individuals, decreased muscle oxygenation gives us essential messages in terms of comorbidities that may be observed in later ages. We hope that our study will raise awareness in young individuals who smoke and the literature.

## Data Availability

The data that support the findings of this study are available from the corresponding author upon reasonable request.
